# Undergraduate technical skills training guided by student tutors – Analysis of tutors' attitudes, tutees' acceptance and learning progress in an innovative teaching model

**DOI:** 10.1186/1472-6920-8-18

**Published:** 2008-04-09

**Authors:** Peter Weyrich, Markus Schrauth, Bernd Kraus, Daniel Habermehl, Nicolai Netzhammer, Stephan Zipfel, Jana Jünger, Reimer Riessen, Christoph Nikendei

**Affiliations:** 1 Department of Internal Medicine IV (Diabetes, Endocrinology, Angiology, Nephrology and Clinical Chemistry), University Hospital of Tübingen, Germany; 2 Department of Internal Medicine II (General Internal and Psychosomatic Medicine), University Hospital of Heidelberg, Germany; 3 Department of Internal Medicine VI (Psychosomatic Medicine and Psychotherapy), University Hospital of Tübingen, Germany; 4 Department of Internal Medicine (Interdisciplinary Intensive Care Unit), University Hospital of Tübingen, Germany

## Abstract

**Background:**

Skills labs provide a sheltered learning environment. As close supervision and individual feedback were proven to be important in ensuring effective skills training, we implemented a cross-year peer tutor system in our skills lab of internal medicine that allowed intense training sessions with small learning groups (3–4 students) taught by one student tutor.

**Methods:**

The expectations, experiences and criticisms of peer tutors regarding the tutor system for undergraduate skills lab training were investigated in the context of a focus group. In addition, tutees' acceptance of this learning model and of their student tutors was evaluated by means of a pre/post web-based survey.

**Results:**

14 voluntary senior students were intensely prepared by consultants for their peer tutor activity. 127 students participated in the project, 66.9% of which responded to the web-based survey (23 topics with help of 6-point Likert scale + free comments). Acceptance was very high (5.69 ± 0.07, mean ± SEM), and self-confidence ratings increased significantly after the intervention for each of the trained skills (average 1.96 ± 0.08, all p < 0.002). Tutors received high global ratings (5.50 ± 0.07) and very positive anonymous individual feedback from participants. 82% of tutees considered the peer teaching model to be sufficient, and a mere 1% expressed the wish for skills training to be provided by faculty staff only. Focus group analyses with tutors revealed 18 different topics, including profit in personal knowledge and personal satisfaction through teaching activities. The ratio of 1:4 tutor/tutees was regarded to be very beneficial for effective feedback, and the personalized online evaluation by tutees to be a strong motivator and helpful for further improvements. The tutors ascribed great importance to the continuous availability of a contact doctor in case of uncertainties.

**Conclusion:**

This study demonstrates that peer teaching in undergraduate technical clinical skills training is feasible and widely accepted among tutees, provided that the tutors receive sufficient training and supervision.

## Background

Skills lab facilities allow structured training of undergraduate medical students for the acquisition of clinical skills in a simulated and sheltered learning environment. Since such skills labs train students using manikins, part-task-trainers or simulators, patient injuries arising from the inexperience of medical trainees can be prevented, thus fulfilling an ethical imperative [1]. The effectiveness of skills labs has been investigated and received much support from several studies employing a variety of methods. Skills lab training improves both physician-patient relationships and patient safety [2,3], increases the frequency of performed skills on the ward [4], leads to higher scores in written skills' tests [5] and to improved OSCE performances in both a longitudinal [6] and a prospective controlled design [7,8]. Training in skills labs may encompass isolated technical clinical procedures, communication skills or the structured acquisition of complex medical algorithms [9]. In a recent meta-analysis, several important factors were identified that facilitate a sustainable learning effect in skills labs. These included repetitive practice, curriculum integration, controlled environment, defined learning goals, simulator validity, close supervision and individual feedback to the learners in order to eliminate errors as early as possible [10].

Peer assisted learning (PAL) is an established learning method which has been practised for centuries across many educational disciplines. A number of different peer teaching methods have been described [11]. While same-year peer teaching implies that students of a similar educational level form a learning group with the goal of coaching one another, cross-year peer teaching encompasses a certain hierarchy based on varying educational levels, meaning that a more advanced student teaches a lower level fellow student [12]. A typical specialty within the field of medicine in which peer teaching systems are implemented into medical education is anatomy, where both same-year [13] and cross-year peer teaching [14] are effective. Cross-year peer teaching is also effective in the areas of communication and nursing skills [15-17].

Some studies addressed the acceptance and efficiency of peer tutor systems in training of clinical examination skills [18,19], revealing that 4^th ^year medical students were equally as effective and accepted in introducing 1^st ^year medical students to the basic physical examination as medical staff members [20]. To our knowledge, the integration of a peer tutoring system in skills lab training of technical skills in internal medicine has not been investigated so far. Furthermore, the number of studies that have investigated the impact of a tutor system on the peer tutors themselves is rather limited [21], with a recent study reporting that the main personal benefit of final year students tutoring 3^rd ^year medical students in clinical examination is their improvement in teaching and clinical skills [22].

The aims of the present study were (a) to investigate whether peer teaching provides a feasible model for undergraduate technical skills training in medical education, (b) to analyze the personal motivations and experiences of student peer tutors, (c) to estimate the learning progress made by the tutees and, finally, (d) to verify whether student tutors became accepted by their tutees as teachers in a skills lab of internal medicine. For this purpose, a focus group analysis of peer tutors was conducted following completion of the project. Positive and negative experiences during the project were analysed, and strategies for future improvements developed. Acceptance of the technical skills training tutor model among the tutees themselves was evaluated by a web-based survey, and self-confidence ratings of tutees were employed as a surrogate parameter of the model's effectiveness.

## Methods

### Definition of learning objectives

In a first step, learning objectives of the training were adapted according to the basic procedural skills required in internal medicine. To this end, a consultant of internal medicine and six 4^th ^year students met in a focus group to assess the training syllabus. An agreement was made with the student tutors that clinical background information on each clinical skill should also be given in the training in order to facilitate an integrative learning environment. The list of compiled technical skills and corresponding exemplary background information is provided in Table 1.

**Table 1:** Learning objectives of peer-guided technical skills training

**Technical skill**	**Theoretical background information (selection)**
Blood pressure measurement	WHO classification of arterial hypertension, 'white coat hypertension', multiple measurements needed for diagnosis of arterial hypertension
Correct skin cleaning and disinfection	Phlegmons, thrombophlebitis, sepsis
Safe syringe disposal	Accidental pinprick and statistics of Hepatitis C and HIV transmission, HIV post-exposition prophylaxis
Blood sampling	Tricks for finding suitable veins, avoidance of haemolysis
Intravenous cannulisation	List of drugs contraindicated for peripheral administration
Intramuscular injection (M. deltoideus)	Guidelines for vaccine application
Intramuscular injection (M. gluteus)	
Intradermal skin test placement	Typical clinical applications such as for example prick testing, tine-test in tuberculosis
Electrocardiogram registration	Indications, problems due to incidental reversal of electrodes

Learning objectives were gathered by consultants and 4^th ^– 5^th ^year medical students following a needs assessment.

### Recruitment, training and supervision of peer tutors

A cross-year teaching system was implemented on account of the fact that only senior students have enough personal clinical experience which was considered to be an indispensable prerequisite for achievement of the learning objectives. Recruitment of 4^th ^– 5^th ^year student tutors occurred on a voluntary basis after a personal interview with the mentoring doctor. Former experiences as a tutor, teacher, team leader, accomplished clerkships as well as the level of motivation served as criteria for selection [23], with tutors receiving a contract and financial compensation from the faculty as student assistants. Consultants of internal medicine were responsible for the training of the peer tutors. Two training sessions were carried out in the skills lab of internal medicine, each lasting three hours. During the training, internal consultants and student tutors discussed the tutors' former clinical clerkship experiences, in particular with respect to the technical skills selected for the syllabus and with the goal of reaching a consensus concerning how each skill should be taught. All procedures were subsequently demonstrated in a step-by-step manner by consultants and repeatedly practised by the tutors. Additional clinical background information was provided for each of the technical skills, such as for example information on the frequency of Hepatitis C or HIV transmission in accidental pinpricks for the learning objective 'safe syringe disposal', or a list of drugs contraindicated for long-term peripheral administration for the learning objective 'intravenous cannulisation'. In addition, a detailed manual was compiled for the course by five experienced clinicians and supplied to the peer tutors as a reference guide. The manual comprised three chapters on each of the procedures, including relevant clinical background information, a checklist containing procedural step-by-step instructions and additional extensive pictorial documentation of each technical skill. The checklists were adapted from the catalogues used at the University of Heidelberg for feedback of role plays in skills labs [24]. Before the manual was distributed among the tutors, five selected peer tutor students were asked to read the manual and to add comments for improving its usability and didactic quality. One consultant was responsible for coordination and acted as a contact doctor for the duration of the project.

### Skills Training

Our Department of Internal Medicine offers three different skills lab trainings, namely a basic course in 3^rd ^year, advanced course in 5^th ^year and refresher course in 6^th ^(final) year of medical education. These courses are dedicated to train mainly procedural skills, as training of communication, interviewing and physical examination skills is provided separately by faculty staff. The students were informed that parts of the basic course syllabus may be subject of regular OSCE assessments at our faculty, following the skills training 6 months later. Participation was obligatory for all 3^rd ^year medical students and the training was carried out in groups of 6 – 8 individuals. These groups were instructed by two peer tutors, resulting in a ratio of one tutor to a maximum of four tutees. Prior to technical skill demonstrations, background information was provided by the tutors for each skill. Tutors taught the background information according to the previous training sessions with the consultants and were instructed to adhere to the tutor manual provided. Following a step-by-step demonstration by the tutors, the tutees were given the opportunity to practise the skills under supervision of the tutors with continuous feedback. The ratio of one tutor to a maximum of four tutees was selected in order to facilitate close supervision of tutees with continuous feedback from tutors. Two skills lab sessions (one week apart) – each lasting three hours – were planned in order to cover the syllabus. In case of uncertainties or questions, it was possible to localize the contact doctor by pager at any time.

### Evaluation of the peer tutor system by trainees

Participants received an online questionnaire via email from an internet-based survey tool designed to attain students' self-confidence directly before and after the training, and their attitudes towards their peer tutors after the second skills lab session. The questionnaire included both quantitative and qualitative questions. A 6-point Likert scale was used for self-confidence rating (1 = I feel very unconfident; 6 = I feel very confident) with respect to each of the technical skills (see table 1) and for the evaluation of the skills training (1 = insufficient; 6 = very good). The questionnaire also included free text fields for personal comments to the peer tutors as well as to the contact doctor. Both tutors and contact doctor had no access to the evaluations until completion of the courses and the focus group. An additional text field was reserved for comments concerning the peer tutor system in general. The online evaluation was provided by Ostrakon™ (Ostrakon Ltd, Tübingen, Germany).

### Focus group analysis of peer tutors

Focus groups in general reveal a broad range of subjective opinions. In light of this, it was decided that this assessment tool should be used to investigate tutors' beliefs and attitudes with regard to the following three standardised questions: (i) what motives did you have in volunteering to work as a peer tutor? Did you have specific expectations or apprehensions? (ii) What experiences did you have with your 'tutees' during your job as peer tutor? Do any especially positive or negative experiences spring to mind? What will you particularly remember in the future? (iii) What possibilities and potential do you see for further development and improvement of student peer-guided undergraduate technical skills lab training? The focus group analysis took place immediately after the three month project, at a point in time when tutors yet had no access to the evaluation results. The session was carried out in accordance with the recommendations of Barbour [25]. Neither the moderator nor the focus group typist was involved in the project or in issues of student examination at the respective faculty. The standardized questions were followed by an open discussion. Protocols recorded by a typist were transcribed and subjected to semantic content analysis.

### Ethical aspects

The ethics committee at the University of Tuebingen waived the requirement for an ethical approval procedure for above described study design.

### Statistical analysis

All data obtained from Likert scale ratings are shown as mean ± standard error of the mean (SEM). Pre/post self-confidence comparisons were carried out using the exact sign test for paired samples, and p-values of < 0.05 were considered to be statistically significant. The software packages JMP (SAS Institute Inc, Cary, NC, USA) and STATISTICA 7.0 (StatSoft Inc., Tulsa, OK, USA) were used for statistical analyses.

## Results

### Sample

All trainees were at the start of their 3^rd ^year of medical education, and had just completed the preclinical section of their degree (n = 127; 22.9 ± 0.6 years; 45/82 male/female). 14 4^th ^– 5^th ^year medical students (8 male, 6 female; mean age: 24.4 ± 0.5 years) were recruited as peer tutors from a remarkably large number of voluntary applicants.

### Acceptance of student peer-guided undergraduate skills lab training

**Figure 1:**
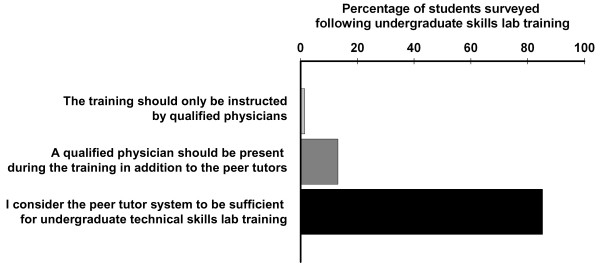
**Tutees' global acceptance of student tutors**. Answers in percentage of surveyed students following peer-guided skills lab training (n = 85).

Of the 127 participating students, 85 (= 66.9%) responded to all questions from the online survey tool. The global quality of the student peer-guided undergraduate skills lab training was rated very highly among the learners (5.69 ± 0.07 on a 6-point Likert scale). Students strongly agreed with the statement that learning objectives were clearly defined by the tutors and that there was enough time to ask their peer tutors questions. They also acknowledged that peer tutors were sufficiently prepared for their teaching activity (ratings in all questions > 5.31; see table 2). Students' ratings of the individual tutors ranged from 4.5 to 6.0, and a global tutor rating of 5.50 ± 0.07 (6.0 = 'very good') was obtained. 85% of trainees considered the peer system to be sufficient for undergraduate skills lab training, 14% expressed the wish for additional teaching by a qualified physician and 1% stated that the training should only be carried out by faculty staff in the future (Fig. 1).

**Table 2:** Evaluation of student tutors

Learning objectives were clearly defined by the peer tutors^a^	5.51 ± 0.09
I had enough opportunities to personally ask questions of my peer tutor^a^	5.73 ± 0.07
My peer tutor was sufficiently trained prior to his/her teaching activity^a^	5.31 ± 0.11
How would you evaluate your personal tutor?^b^	5.50 ± 0.07
Global quality of peer-guided skills training^b^	5.69 ± 0.07

^a^6-point Likert scale: 1 = I strongly disagree; 6 = I strongly agree (mean ± SEM).

^b^6-point Likert scale: 1 = insufficient, 6 = very good (mean ± SEM).

Acceptance of student peer-guided skills lab training by trainees (n = 85).

Free text fields contained a total of 32 personal comments to the student tutors, 30 comments to the contact doctor and 7 proposals for future improvement of the skills training syllabus. Participants' remarks to the peer tutors were markedly positive with respect to their competence and in particular their patience during the skills training. Many students mentioned that they felt less inhibited asking questions of their tutors than of faculty members, who were generally regarded to be too busy to spend the time needed for detailed explanations. Content analysis of items directed to the contact consultant revealed that the students were highly satisfied with both the general concept and the defined learning objectives of the training. Some students suggested that training in basic skills should in general be carried out by student tutors and that faculty members should act more as a supervisor for the tutors instead of teaching the students themselves. One recurrently mentioned proposal for further improvement was an increase in the number of opportunities for practising skills, possibly under supervision of a single tutor in case of further questions.

### Qualitative focus group analyses of peer tutors

13 of the 14 tutors participated in the focus group discussion. Main motives for their tutoring activity were participating in a relevant medical education project, the expected increase in own expertise in technical skills and the personal satisfaction linked with the teaching activity itself. Three students mentioned that they were concerned about problems of competence or authority before starting their tutoring activity. Among the positive experiences gained as a student tutor, the most frequently mentioned aspect was the pleasant learning atmosphere and the general delight in mutual learning that was facilitated by the 3:1 – 4:1 ratio of students to tutor. Tutors appreciated the constant availability of a contact consultant in case of problems or questions. Three tutors acknowledged that their tutoring role had helped them to overcome previous anxiety and self-doubt. Negative experiences were also reported: tutors complained of a pretentious attitude of some students with former nursing or paramedic qualifications having prior technical skills knowledge at their disposal, or at least pretending to do so. Though not a frequent problem, this previous knowledge has resulted in inhomogeneity among the tutees and made appropriate teaching difficult in sporadic cases. Besides changes in the local infrastructure, more time for participants to practice was itemized as a measure for the improvement of training outcome. Student tutors proposed that a tutor should be present during such voluntary exercise units. Two tutors missed an additional training in didactics before the project. An interesting statement was made by two other tutors concerning the financial compensation of the tutors: while they agreed that this reward adds professionalism to the project, they also mentioned that compensation should not be too high in order to avoid tutor applicants with exclusively financial interests and no personal identification with their upcoming tutor activity. All items brought up in the focus group discussion are listed in detail in Table 3.

**Table 3:** Focus group analysis of student tutors

**MOTIVATIONS AND EXPECTATIONS**	**APPREHENSIONS**	
Participation in an innovative and relevant medical education project (7)^a^	Concernment about problems of expertise, qualification and competence (3)	**BEFORE**
Broadening of personal competence in technical skills (7)		
Enjoyment and personal satisfaction through teaching activities (5)		

**TUTORS' POSITIVE EXPERIENCES**	**TUTORS' NEGATIVE EXPERIENCES**	

Pleasant learning atmosphere (7)	Pretentious attitude of 'know-it-all' students (4)	**DURING**
Continuous presence of a contact doctor (7)		
Students' sense of achievement, gratefulness and positive feedback (5)		
Delight in mutual learning (4)	Inhomogeneity of participants' prior knowledge/experience in technical skills (3)	
Beneficial proportion of tutors relative to the number of students (4)	Varying learning motivation among participants (2)	
Overcoming of tutors' and students' anxiety and self doubts (3)		

**PROPOSALS FOR FUTURE IMPROVEMENTS**	**MAJOR POINTS OF DISCUSSION**	

Changes in local infrastructure/premises (4)	Financial compensation: should be paid as gratuity to give a more professional character to tutors' teaching activities, but should not be too high in order to avoid peer tutor applications based purely on financial interests (2)	**AFTER**
More time for participants to practice further (3)		
Additional training of tutors in didactics (2)		
'Sorting' of participants according to individual prior knowledge in order to achieve more homogeneity among learners (1)		

Focus group analysis was carried out with 13 peer tutors. Items are arranged according to the three introductory questions of the focus group, the time point (before, during and after tutor activity; right column) and frequencies of reference.

### Learners' self-confidence following peer-guided technical skills lab training

By means of the online survey tool, participants provided their pre/post self-confidence ratings for each skill taught before and after the skills lab training. While participants felt confident measuring blood pressure and almost confident regarding correct skin disinfection and the safe disposal of used syringes even prior to training, they felt unconfident with respect to more advanced skills such as for example drawing blood, giving injections, cannulisation and ECG registration. Self-confidence rating improved significantly for all procedures included in the skills lab training. For the six advanced skills, students improved on average 1.96 ± 0.08 points on the 6-point Likert scale (see Table 4).

**Table 4:** Self-confidence of tutees

**Clinical skill**	**before training**	**after training**	p*
Blood pressure measurement	5.13 ± 0.13^a^	5.45 ± 0.09	<0.0001
Correct skin cleaning and disinfection	4.56 ± 0.14	5.52 ± 0.08	<0.0001
Safe syringe disposal	4.76 ± 0.16	5.49 ± 0.09	<0.0001
Blood sampling	2.99 ± 0.19	4.79 ± 0.10	<0.0001
Intravenous cannulisation	2.35 ± 0.19	4.28 ± 0.12	<0.0001
Intramuscular injection (M. deltoideus)	2.36 ± 0.19	4.41 ± 0.14	<0.0001
Intramuscular injection (M. gluteus)	2.45 ± 0.19	4.67 ± 0.12	<0.0001
Intradermal skin test placement	2.30 ± 0.21	4.35 ± 0.17	<0.0001
Electrocardiogram registration	2.61 ± 0.21	4.32 ± 0.14	<0.0001

^a^6-point Likert scale: 1 = I feel very unconfident; 6 = I feel very confident (mean ± SEM).

*Exact sign test for paired samples.

Self-confidence of 3rd year trainees (n = 85) before and after tutor-based skills lab training.

## Discussion

Student tutor programs are implemented in many curricula across both medical schools and other disciplines of higher education [11]. Despite the wide distribution of this teaching model, most programs are rather informal and not evaluated to the same extent as seminars taught by regular faculty members. So far, only a limited number of studies have investigated the effectiveness and acceptance of a student tutor program in clinical skills training. Peer teaching models have, for example, been described for communication skills [15,16,21] and physical examination skills training [18,22,26]. This is the first study to show that cross-year peer tutoring for undergraduate procedural skills training in medical education is feasible and, in addition, also very well accepted among tutees.

A large majority of the tutees participating in the study considered the student tutor system to be sufficient, with only every eighth student wishing for the additional presence of faculty staff. The open comments of the participating students directed to the contact consultant predominantly underscored the general advantages of a beneficial learning partnership with the tutors. These advantages included more time for individual feedback, greater acceptance by their tutors who were at the same learning stage not so very long ago and the tutors' profound understanding and personal identification with students' typical struggles, recently been identified as an important general advantage of PAL in other studies [27,28]. The self-confidence survey of tutees revealed remarkable progress in all taught skills, and tutees felt less inhibition when asking questions of tutors rather than of established medical staff. However, it has to be mentioned that especially the latter advantage of PAL also may imply the drawback that contact time between students and medical doctors may decrease significantly with implementation of PAL. Therefore, PAL always should be used in balance with traditional education by established staff.

A further aim of the study was to investigate tutors' attitudes and experiences by means of a focus group. The expected gain in personal knowledge about technical skills constituted students' main motive in becoming a tutor. Students were especially convinced that mastering the selected syllabus as a tutor would be relevant for their future professional career. Given that engaging in tutor activity has been shown to result in significantly greater content-specific and generalized cognitive profit than being tutored [29], this expectation would appear to be justified. Positive aspects clearly dominated the experiences of tutors during the project. All tutors agreed that the pleasure of being accepted as a teacher in addition to the observed gain in their group leadership qualities enhanced personal motivation. They considered this profit to far exceed any financial compensation and would act as a tutor again even without payment. We nonetheless continue to contract all tutors, not only for legal reasons, but also in the belief that such an approach reinforces motivation and responsibility.

In our skills lab, only senior students act as tutors. We doubt the effectiveness of same-year peer teaching (mutual coaching by students of a similar level) in the area of technical skills, on account of the considerable importance of previous clinical experience with real patients. It has been shown that peer teaching in surgical skills training with novices can even worsen the training outcome [30]. Thus, quality management in peer teaching with thorough and structured training of tutors prior to teaching activity is indispensable. For this reason, the tutors are trained in small groups in the skills lab before embarking upon their teaching activity, and a detailed and intensely illustrated manual with step-by-step checklists is provided as a reference guide. These measures are designed to ensure the necessary quality of skills teaching and to prevent tutors encountering problems with respect to competence when dealing with students who have previous knowledge. In addition, our tutors are individually and anonymously evaluated by the students participating in the training. This feedback further will help them to improve their didactic skills for future teaching activities. Following the focus group analysis, the issues provided by the tutors were used as a basis for several debriefing sessions with consultants. In these debriefing meetings, potential strategies for dealing with those problems which had arisen in the skills labs, for example the difficult attitude of participants with previous knowledge, were developed. In the meantime, we additionally support the tutors with a seminar for didactics to ensure long-term sustainability of the peer teaching quality in undergraduate skills lab training.

The close and competent supervision of learners is of pivotal importance in ensuring high quality technical skills training, since the performance of skills without supervision or feedback may serve to increase personal confidence, but not competence [31]. In light of this, remarkable efforts must be made to adequately train student tutors and to maintain this model in our curriculum. Questions concerning the cost-effectiveness and profitability of student tutor-guided technical skills training may thus arise. It should be pointed out, however, that the majority of our tutors decided to continue their teaching activity in the skills lab and that these experienced tutors, in addition to established faculty staff, are now involved in training novice tutors. As senior tutors share their experiences with beginning tutors in a dyadic tutor team, the setting of our skills lab training may represent the beginning of a typical mentor learning pyramid [32].

### Study limitations and future research

#### Recruitment of student tutors

It is evident that motivation of the student tutors represents an important variable for the outcome and quality of peer teaching programmes. In this study, student tutors were recruited on a voluntary basis and, therefore, may not be representative for all students. As the number of voluntary applications for student skills lab tutor jobs always markedly exceeded the number of available positions in the ensuing three semesters after the study, it would be an interesting goal of future studies to define criteria that may help to identify tutors with good teaching quality and acceptance among the tutees.

#### Study design

It has to be mentioned that the assessment of the student tutors' teaching quality is based on self-confidence measures in the web-based survey among the tutees. First of all, the limited response rate (66.9%) to the web-based survey tool may contribute to a selection bias. In addition, neither subjective perceptions of a pleasant and constructive learning environment nor students' self-confidence ratings imperatively reflect objective competence in their clinical skills [33,34]. To definitely assess the quality of undergraduate technical skills training by student tutors, an objective assessment tool [35] following the skills lab training units has to be employed in future studies. We also cannot rule out that the information about a potential future OSCE assessment of the trained skills may have affected the students' attitudes and motivation in the sense of "assessment drives learning" [36].

## Conclusion

In summary, the present study demonstrates that peer teaching for clinical skills training is feasible, well accepted and perceived to be effective in the subjective self-confidence ratings of tutees. Although tutors were concerned about problems of expertise and competence before their teaching activity, the tutees' evaluation of their personal tutor was generally very good. From the tutors' perspective, only learners with prior knowledge (former medical education in nursing etc.) sometimes were reported to be problematic, while a pleasant learning atmosphere was perceived by all other participating students. Positive personal feedback for their tutoring activity, both directly during the training sessions and through the web-based survey tool after the completion of focus groups, was reported to be a strong motivating factor. Sufficient tutor training and preparation is crucial for the success of peer teaching models, and the continuous availability of a contact doctor is important to minimize tutors' uncertainties. Further studies are now needed to investigate the effectiveness of this teaching model, employing established assessments tools, as e. g. objective structured clinical exams.

## Competing interests

The author(s) declare that they have no competing interests.

## Authors' contributions

PW is responsible for the skills lab of Internal Medicine at the University in Tuebingen, made substantial contributions to both study design and data analysis, acted as teaching and contact doctor for the student tutors, prepared all tables/figures and drafted the manuscript. MS has carried out the focus group analysis and participated in drafting the manuscript. BK contributed conceptual ideas for the study design and proof-read the written tutor manual. DH is responsible for continuous evaluation of medical education at the University of Tübingen and carried out the web-based survey of participants. NN acted as a student tutor during the project and cared of many organizational details in the skills lab. SZ placed parts of his grant at disposal for realization of the project and contributed to the study design. RR acted as a trainer for the student tutors. JJ and CN contributed central conceptual ideas to the study design, and CN intensely contributed to the preparation of the manuscript. All authors read and approved the final manuscript.

## Pre-publication history

The pre-publication history for this paper can be accessed here:


